# More fat, less migration: breast density as a predictor of sentinel lymph node non-visualization in breast cancer

**DOI:** 10.1186/s13550-021-00848-w

**Published:** 2021-10-29

**Authors:** Elske Quak, Grégoire Braux, Kathleen Weyts, Charline Lasnon

**Affiliations:** 1 Nuclear Medicine Department, Comprehensive Cancer Centre François Baclesse, UNICANCER, Avenue Général Harris, 14076 Caen Cedex 5, France; 2 Radiology Department, Caen University Hospital, 14000 Caen, France; 3 UNICAEN, INSERM 1086 ANTICIPE, Normandy University, 14000 Caen, France

## Dear Editor,

We carefully read the paper written by Chahid et al. entitled “Risk Factors for Non-Visualization of the Sentinel Lymph Node on Lymphoscintigraphy in Breast Cancer Patients” recently published in EJNMMI research [1]. After investigation of a large dataset of patients and application of multivariable analysis, this team found that the ages of ≥ 70 years (*P* < 0.001; OR: 2.27; 95% CI: 1.46–3.53), body mass indexes (BMIs) of ≥ 30 kg/m^2^ (*P* = 0.031; OR: 1.48; 95% CI: 1.04–2.12), and non-palpable tumors (*P* = 0.004; OR: 1.54; 95% CI: 1.15–2.07) were independent predictors of sentinel lymph node (SLN) non-visualization. Therefore, it was concluded that SLN lymphoscintigraphy is a very robust technique that does not depend on the experience of the preparer or administrator of the radiotracer which is indisputable. It is worth noticing that these findings were concordant with those of some previous studies [2–4].

In a comprehensive analysis of breast density distribution of 821 Finnish women with breast cancer [5], it was observed that breast density categories had an association with age and BMI. Indeed, the older and more obese patients were mostly represented in the lower-density categories. In daily clinical practice, when confronted with an elderly female with large and fatty breasts for the SLN procedure, the assumption that the odds of non-visualization are high is frequently right. Therefore, we asked ourselves whether breast density, rather than age and BMI, could be the major variable to take into account for the prediction of SLN non-visualization.

To verify this hypothesis, we randomly selected 210 out of the 569 patients addressed to our unit for breast SLN lymphoscintigraphy in 2018. Their data concerning age, BMI, and breast density were available for 184 patients. The palpability of tumors, which was also identified as an independent predictor in the study of Chahid et al., was not recorded. Breast density of the patients was determined by an experienced radiologist according to the Breast Imaging-Reporting and Data System atlas on mammography and/or breast magnetic resonance imaging (MRI) when available (*n* = 81). Based on their breast density, they were divided into categories one (almost entirely fatty, *n* = 19, 10.3%), two (scattered densities, *n* = 115, 62.5%), and three/four (heterogeneous/extremely dense, *n* = 50, 27.2%).

**Fig. 1:**
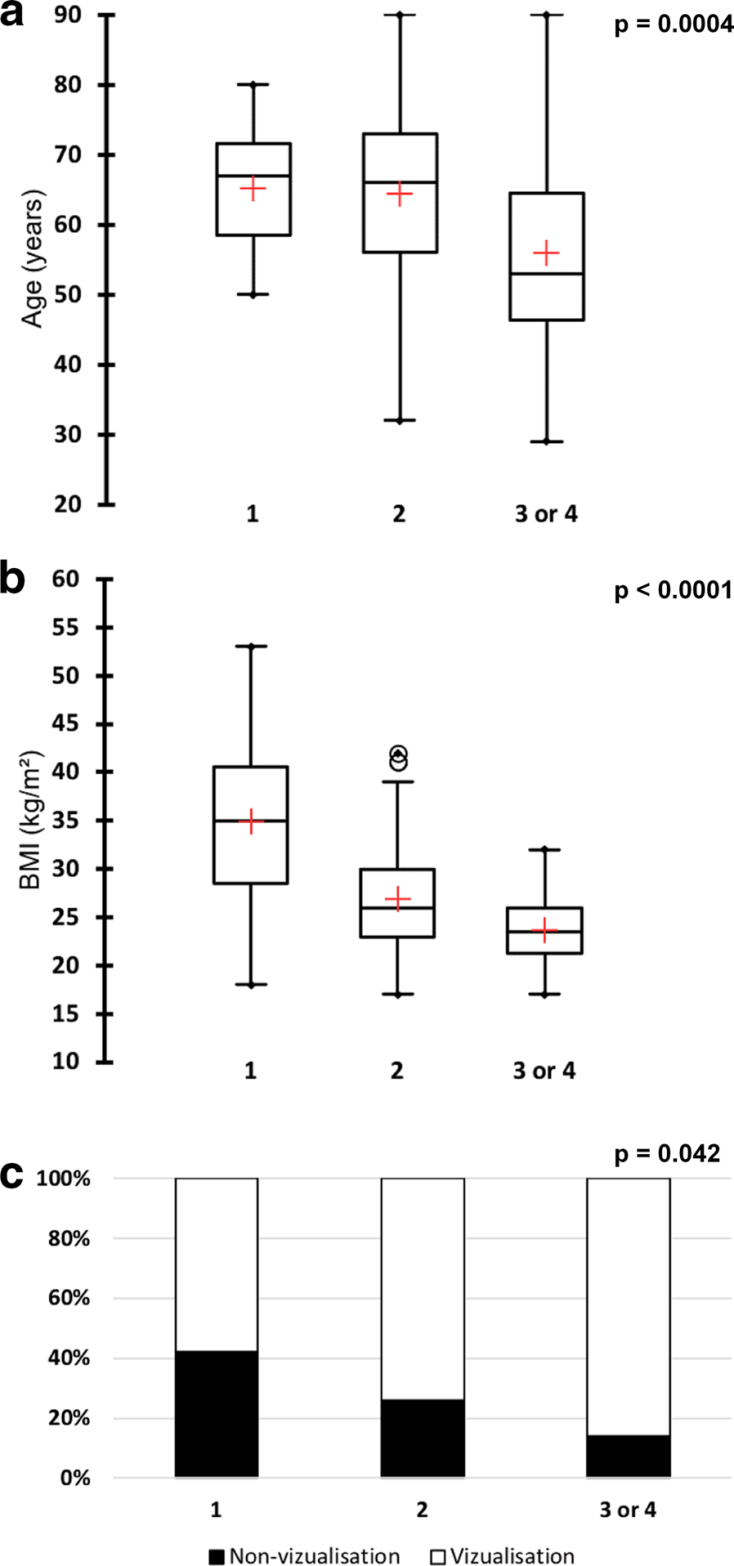
Comparison of age (**a**) and BMI (**b**) of breast density categories using Kruskal-Wallis tests and comparison of the proportions of sentinel lymph node non-visualization cases among breast density categories (**c**) using a Fischer exact test. BMI: Body Mass Index. Category 1: almost entirely fatty. Category 2: scattered densities. Category 3 or 4: heterogeneous or extremely dense

In accordance with the previously described Finnish study, we found that breast density was significantly associated with both age (*P* = 0.0004) and BMI (*P* < 0.0001); accordingly, lower densities were observed in older and heavier patients (Fig. 1a, b). Even if trends were observed, Mann–Whitney tests showed that the age and BMI of patients for whom the SLN was not visualized were not different from those of other patients: 65.2 ± 11.1 years versus 61.2 ± 13.0 years, respectively (*P* = 0.08) and 28.3 ± 6.7 kg/m^2^ versus 26.4 ± 5.9 kg/m^2^, respectively (*P* = 0.06). Moreover, we observed a significant association between breast density and SLN detectability; accordingly, patients with lower breast densities had low SLN detectability (Fig. 1c). In other words, lymphatic drainage was less visible in fattier breasts.

The rarity of lymphatic capillaries within human subcutaneous adipose tissue, as recently reported by Redonda et al. [6, 7], supports our finding that breast density plays an important role in the success of the lymphoscintigraphy protocol.

With this letter, we would like to draw attention to the correlation between breast density and SLN non-visualization, in addition to the findings reported by Chahid et al. The time seems right to launch a debate on the potential use of breast density (easily determined by mammography and breast MRI that patients undergo as part of their baseline assessment) for the eventual development of SLN protocol adaptation.

## Data Availability

The data supporting the conclusions of this article will be made available by the authors, upon reasonable request.
